# Simulation-based assessment of digital twin systems for immunisation

**DOI:** 10.3389/fdgth.2025.1603550

**Published:** 2025-08-22

**Authors:** Leonardo de Oliveira El-Warrak, Claudio Miceli de Farias, Victor Hugo Dias Macedo De Azevedo Costa

**Affiliations:** ^1^ COPPE - Graduate School and Research in Engineering, Federal University of Rio de Janeiro (UFRJ), Rio de Janeiro, Brazil; ^2^ FEN - Graduate School in Engineering, State University of Rio de Janeiro (UERJ), Rio de Janeiro, Brazil

**Keywords:** immunisation, digital twin, vaccines, TwinVax, simulation, IoT

## Abstract

**Background:**

This paper presents the application of simulation to assess the functionality of a proposed Digital Twin (DT) architecture for immunisation services in primary healthcare centres. The solution is based on Industry 4.0 concepts and technologies, such as IoT, machine learning, and cloud computing, and adheres to the ISO 23247 standard.

**Methods:**

The system modelling is carried out using the Unified Modelling Language (UML) to define the workflows and processes involved, including vaccine storage temperature monitoring and population vaccination status tracking. The proposed architecture is structured into four domains: observable elements/entities, data collection and device control, digital twin platform, and user domain. To validate the system's performance and feasibility, simulations are conducted using SimPy, enabling the evaluation of its response under various operational scenarios.

**Results:**

The system facilitates the storage, monitoring, and visualisation of data related to the thermal conditions of ice-lined refrigerators (ILR) and thermal boxes. Additionally, it analyses patient vaccination coverage based on the official immunisation schedule. The key benefits include optimising vaccine storage conditions, reducing dose wastage, continuously monitoring immunisation coverage, and supporting strategic vaccination planning.

**Conclusion:**

The paper discusses the future impacts of this approach on immunisation management and its scalability for diverse public health contexts. By leveraging advanced technologies and simulation, this digital twin framework aims to improve the performance and overall impact of immunization services.

## 1 Introduction

Global health has faced unprecedented challenges in recent decades, with the COVID-19 pandemic pushing healthcare systems to their operational limits. This crisis exposed critical vulnerabilities, including an exponential surge in patient volume, shortages of essential resources, technical gaps in healthcare workforce preparedness, and an overload of fragmented or inaccurate information that impeded effective decision-making. Despite immunisation being one of the most impactful public health interventions, the pandemic severely disrupted routine vaccination programs worldwide, particularly in 2020 and 2021, causing significant setbacks in disease prevention efforts (1–3).

Faced with persistent challenges in controlling vaccine-preventable diseases, technological advances have emerged as a vital ally. New technologies offer a solid basis for faster and more effective responses during health crises, while simultaneously improving the general health of populations under normal circumstances. By integrating these solutions into health systems, it is possible to enable a more precise and consistent approach by health teams, improve resource allocation, and strengthen the health response capacity of healthcare systems (4). Vaccination remains the cornerstone of disease prevention, offering robust protection against vaccine-preventable illnesses without the risks associated with natural infection. It not only shields individuals from severe disease outcomes but also curtails the spread of pathogens within communities. Achieving high vaccination coverage fosters herd immunity, effectively interrupting disease transmission chains. Beyond health outcomes, vaccination programs yield extensive societal benefits by reducing healthcare burdens, mitigating economic losses, and enhancing community well-being (5–7).

The healthcare sector is a dynamic landscape of technological innovation, consistently integrating new tools to enhance service delivery and patient outcomes (8, 9). Vaccines, however, are sensitive biological products that require stringent temperature control to maintain their efficacy. Failures in cold chain management—from storage to transportation—remain a leading cause of vaccine spoilage and wastage. In this context, advanced technologies such as the Internet of Things (IoT) and Digital Twins (DT) offer transformative potential. These technologies enhance real-time monitoring, predictive maintenance, and data-driven decision-making, particularly in settings with complex operational challenges.

This paper addresses two critical issues in immunisation programs: (i) vaccine loss due to inadequate temperature control in storage equipment, such as Ice-Lined Refrigerators (ILRs) and thermal boxes, and (ii) suboptimal vaccination coverage in specific target populations. To tackle these challenges, we propose a conceptual framework for a Digital Twin (DT) tailored to support immunisation services within primary healthcare settings. Through simulation, we aim to demonstrate how this DT—referred to as TwinVax—can optimise vaccine storage conditions via real-time temperature monitoring and enhance immunisation coverage through dynamic data analytics, ultimately supporting timely and evidence-based public health decision-making.

The originality of this study lies in the application of Digital Twin technology specifically to immunisation services in primary healthcare—an area that remains largely unexplored in current literature. While many DT initiatives focus on hospital settings or clinical care, there is a notable lack of practical implementations addressing routine vaccination services, particularly in low-resource environments. This paper contributes by presenting a structured and operational DT architecture, based on the ISO 23247 framework, that integrates cold chain monitoring with patient vaccination tracking. Furthermore, the simulation-based validation using real demographic data provides insights into the feasibility and potential impact of such systems. This combination of technological integration and practical assessment helps fill an important gap in public health innovation.

## 2 Related studies

Technological innovations play a central role in shaping health systems, influencing how services are delivered and the outcomes achieved. These innovations encompass a wide range of technologies used for prevention, diagnosis, treatment, and rehabilitation, including vaccines, diagnostic kits, medications, medical equipment, and procedures. The continuous advancement of technologies such as the Internet of Things (IoT), Artificial Intelligence (AI), Machine Learning (ML), 5G, and Big Data has enabled real-time data collection, processing, and storage, fostering new opportunities for healthcare management.

Chaudhari, Gangane, and Lahe (2021) (10) highlight how Digital Twin (DT) technology enhances digital health monitoring within Industry 4.0 concepts, describing DTs as real-time virtual replicas of physical objects that enable continuous health tracking and predictive analytics through IoT and AI integration. Katsoulakis et al. (2024) (11) explore DT applications in healthcare, emphasizing their role in personalizing treatments and improving patient outcomes through real-time data monitoring and computational models. Similarly, Björnsson et al. (2019) (12) discuss DTs as tools for personalized medicine, using patient-specific biological, clinical, and behavioral data to simulate and predict treatment responses.

Stahlberg et al. (2022) (13) examine predictive DTs for cancer patients, focusing on integrating patient data, AI, and computational models to simulate disease progression. The study highlights challenges related to data interoperability, model validation, and ethical concerns, while reinforcing DTs' potential to enhance clinical decision-making in oncology. Popa et al. (2021) (14) analyze DTs from a socioethical perspective, recognizing their benefits in personalized care and decision support but also addressing concerns around data privacy, patient autonomy, and biases in predictive models.

Sahal et al. (2022) (15) investigate the role of emerging technologies—including DTs, blockchain, IoT, and AI—in managing pandemic crises. They propose a blockchain-based framework for decentralized epidemic alerts, stressing the need for secure, real-time data exchange to support COVID-19 response efforts. El-Warrak and Miceli (2024) (16) categorize DT applications in healthcare into clinical and operational domains, covering personalized care, simulation of biological structures, process optimization, and resource management. Despite challenges related to data integration and privacy, DTs show great potential to improve healthcare quality, remote monitoring, prevention, and decision-making.

Uddin et al. (2016) (17) report on an mHealth intervention in Bangladesh using the “mTika” app to improve vaccination rates in hard-to-reach populations. The initiative significantly increased coverage through electronic birth registration and vaccination reminders, demonstrating mHealth's effectiveness despite challenges like data standardization and limited follow-up capacity. Demsash et al. (2023) (18) apply ML algorithms to predict childhood vaccination coverage in Ethiopia, identifying key predictors such as maternal education and healthcare access, though limitations in statistical interpretability were noted.

In Brazil, Ribeiro (2022) (19) proposes a DT architecture to modernize the National Vaccination Plan, enhancing resource management through real-time simulations. In a subsequent study, Ribeiro (2023) (20) introduces a maturity model to assess public healthcare units' readiness for DT implementation, identifying critical factors such as information security, logistics, and organizational management.

The literature review reveals key insights into DT applications in healthcare. Most studies remain theoretical, focusing on opportunities and challenges with limited real-world implementations for evaluation. Some works address Digital Shadows or Digital Models, lacking real-time data exchange, which limits the exploration of DTs' full potential, such as influencing physical systems through intelligent analysis (21). Additionally, DT studies often focus on isolated assets without considering the broader healthcare ecosystem (21, 22). Many rely on simulations disconnected from real systems, neglecting aspects of integration, interoperability, and human involvement (10, 23–25). Finally, while DT functionalities are widely discussed, concrete architectures or methodologies for DT implementation in Primary Health Care are still scarce.

## 3 Methods

### 3.1 Modelling the digital twin in immunisation

The incorporation of digital models as an integral part of studying and developing physical objects is well-established. Virtual prototyping combines digital technology with engineering and design principles, enabling the efficient and precise creation and refinement of physical products and systems. The ability to virtually simulate and analyse objects before physical production has proven invaluable across various sectors, including manufacturing, engineering, architecture, and medicine. This approach offers benefits such as time savings, cost reductions, and optimized product performance and functionality (4, 24).

A model is a representation of a real system, used to conduct simulation studies. To ensure the quality of information derived from the simulation, all elements relevant to capturing essential data from the real system must be incorporated (26, 27). The use of Discrete Event Simulation (DES) in this study is justified by the nature of the data required for modelling the digital twin. DES operates with discrete values that change at specific points in time, allowing clear transitions between states. Additionally, in DES, all data, entities, and activities are identifiable once the model is finalised, enabling a chronological understanding of events.

The core of a DT consists of virtual models, making the development of high-precision digital representations essential. These models must accurately capture the physical properties, behaviours, and governing rules of the real object. The creation of a digital twin involves two primary aspects: (i) developing the processes and information requirements of the DT throughout the product life cycle—from asset design to real-world deployment and maintenance; and (ii) implementing the enabling technology to integrate the physical asset with its digital counterpart. This integration ensures real-time sensor data flow, along with operational and transactional information from the organization's core systems, as outlined in a conceptual architecture.

A digital twin is characterized by four key features: Modelling and Simulation, Real-time Data Integration, Analysis and Optimization, and Insights and Action. Modelling provides a detailed representation of the physical system, encompassing attributes such as mechanical, electrical, and operational properties. Simulation allows testing under diverse conditions to predict behaviour in real-world scenarios. Real-time data integration, powered by IoT sensors, enhances simulation accuracy, and enables early issue detection. Additionally, advanced analytics and machine learning techniques can uncover patterns, predict failures, and suggest improvements.

Real-time or near-real-time physical data updates are critical for refining virtual models and accurately simulating physical processes and their evolution. The network plays a vital role in connecting the physical object (PO) to its virtual representation (VO), enabling real-time data exchange. This connection supports bidirectional communication, where data from the PO updates the virtual model, and insights from the virtual model inform decisions in the physical system. Communication between PO and VO typically involves three stages (28): (i) collecting data through direct measurement of physical conditions, (ii) processing and interpreting data at the appropriate level of abstraction, and (iii) updating system states with integrated data from multiple sources. The interface linking the real process to the DT, represented by the simulation model, varies depending on the characteristics of the simulation software and the connectivity capabilities of the physical system.

A two-phase approach can be employed for predictive modelling in health service management. The first phase involves offline model development, utilizing Machine Learning (ML) and Deep Learning (DL) techniques, such as classifiers, to train the model with historical data from the DT. In this controlled environment, accuracy is improved before real-time deployment. The second phase consists of deploying the trained model online, closer to the data source, to minimize latency and optimize performance. By leveraging real-time streaming data, the model can quickly detect potential risks, enabling rapid responses and necessary adjustments. This two-phase approach integrates extensive historical data analysis with the agility of real-time processing, ensuring a reliable predictive model for proactive health service management. Optimisation is achieved by refining parameters in the digital model and implementing best practices in the physical system. Insights generated by the DT facilitate continuous improvements in healthcare service delivery (29, 30).

Process modelling and simulation are invaluable tools across multiple domains. Recent advances in Industry 4.0, Big Data, IoT, and Sensor Technology have expanded their application in Digital Twins (DT). In this context, process models have evolved from passive tools for hypothesis testing into active components of operational systems. With efficient infrastructures and advanced algorithms, these models can monitor and reflect real-time system states while autonomously executing corrective actions when necessary.

The methodology presented builds upon the framework introduced in (31), originally designed for dynamic modelling in discrete industrial processes. Given the specific needs of immunisation systems—such as vaccine cold chain management and coverage monitoring—this approach has been adapted for healthcare, enabling digital twin applications in immunisation services. The goal is to optimise vaccine storage temperature monitoring and enhance vaccination coverage assessment.

Digital modelling consolidates critical information into a computational environment, allowing analysis and prediction of issues such as temperature fluctuations, vaccine loss risks, and gaps in immunisation coverage. While static digital models do not perform real-time physical simulations, they provide a robust foundation for identifying trends, assessing risks, and supporting decision-making processes. The static models within the DT for immunisation are utilized to evaluate cold chain integrity and predict the potential impacts of storage system failures. This includes cross-referencing historical temperature data with distribution patterns to detect risks that may compromise vaccine efficacy. Additionally, digital modelling supports continuous assessment of vaccination coverage, enabling targeted immunisation campaign planning.

Although static digital models do not capture real-time environmental changes, their continuous updates with sensor data and vaccination records enhance predictive accuracy and risk identification. By integrating data-driven insights into immunisation management, the DT strengthens proactive decision-making and supports the resilience of vaccination programs.

**Figure 1:**
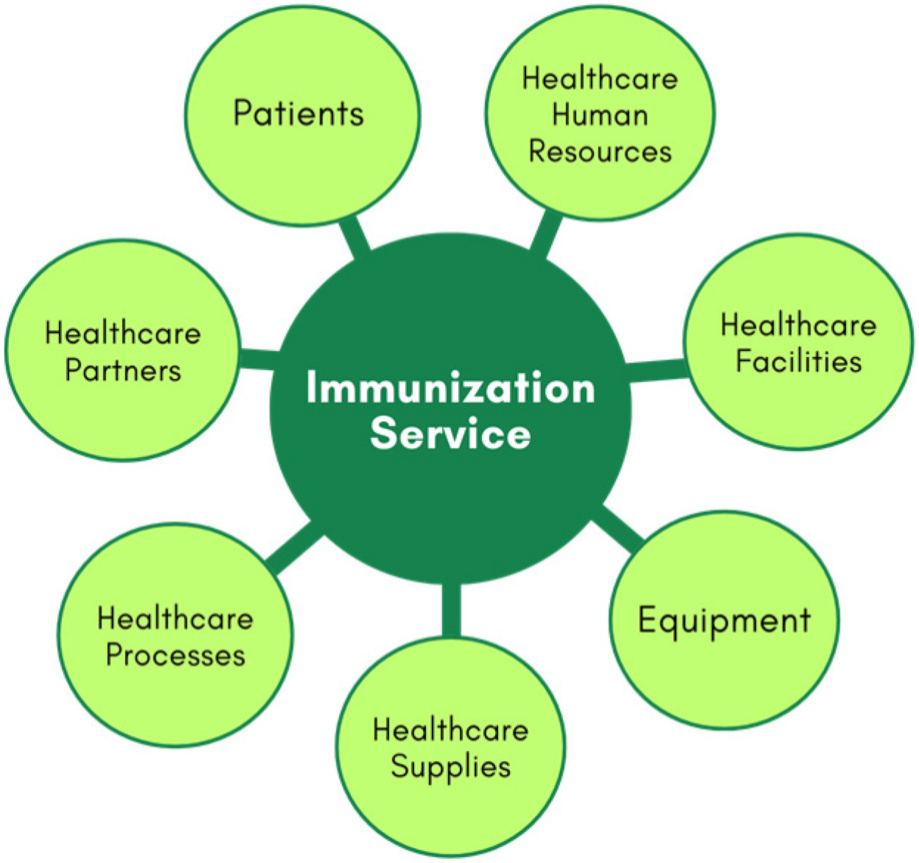
Main components in healthcare process.

For modelling, immunisation services in Primary Healthcare Centre can be divided into seven main components or entities, as illustrated in Figure 1. These components include patients, health human resources, facilities, equipment, health supplies, processes, and partners (32).

The patients' component includes various types of patients, categorized by age groups, health histories, and specific needs, such as those with acute and chronic diseases, disabilities, or immunological risks. The healthcare human resources encompass nurses, technicians, and operational staff. Healthcare facilities cover the immunization room, waiting area, and staff offices. Equipment pertains to all medical devices, IT infrastructure, and furniture. Healthcare supplies are divided into physical and service supplies. Physical supplies include vaccines, medications, drugs, lab materials, cleaning supplies, treatment materials, and other essentials for maintaining healthcare facilities and equipment. Service supplies consist of crucial services received from partners, such as maintenance for medical equipment, catering for staff, patients, and visitors, and utilities like energy and water. Processes include procedures for treating patients with immunobiologicals, managing medical emergencies, organising the vaccine room, staff scheduling, recording information in systems, inventory monitoring of vaccines, supply chain management, workflow optimization, and other operational processes. Partners include suppliers of equipment and consumables, hospitals, specialized healthcare centres, and others.

Digital Twins can be created for all these components. They use data from healthcare facilities, equipment, processes, patients with various needs, supplies, and partners, compiling real-time information from sensors, health information systems, such as electronic medical records (EMR), electronic health records (EHR) and other sources to create digital replicas. For example, digital counterparts can be developed for healthcare facilities such as x-ray rooms and other healthcare processes such as treatment and logistics processes.

On the other hand, creating DTs of patients presents one of the most complex challenges in healthcare due to the need to represent diverse patient characteristics such as age, gender, health history, current health status, and healthcare needs. Studies such as those by (33) have explored the main design requirements and enabling technologies of digital patient twins, as well as the technical challenges present. The complexity involved stems from multiple levels of abstraction, different types of patients, numerous environmental factors, and continuous and rapid changes in healthcare data. Patient digital twins can be developed with varying levels of detail depending on their purpose. For example, refined models can reflect real-time health and environmental information from individual patients, supporting personalized medical services. For most applications in immunization services, these detailed individual models are extremely valid. However, even if there is no detailed health analysis, the immunization service also benefits from an abstract view of aggregated patient data to support high-level decision-making, improving overall efficiency, quality, access, and the cost-effectiveness of vaccination. This model comprises (i) a patient information database populated with clinical data from multiple sources; (ii) a cloud computing platform; (iii) traceability systems using AI; and (iv) blockchain technology. Other components, such as human resources, facilities, and equipment, are less complex and can be generalized based on their specific characteristics. For instance, a digital twin of a nurse would focus on their schedule, work location, and skills, rather than individual traits. Similarly, digital twins for facilities and equipment are relatively static and can be periodically updated as needed.

This study will focus on three components of the represented system: the equipment used for vaccine storage, the vaccines themselves, and the patients. For the equipment entity, the monitoring of operational conditions will be based on the temperature attribute. In the case of the vaccine entity, monitoring will be conducted based on the type and number of doses, while for patients, it will pertain to their vaccination schedule and history. The digital twin will function by providing alerts regarding variations in the ideal temperature conditions for storage that may jeopardize the vaccine's efficacy, as well as alerts for timely vaccination needs for patients.

Furthermore, the Digital Twin should propose scenario analyses for individuals and/or groups who may delay or miss certain vaccinations. Additionally, considering the vaccination needs according to the vaccination schedule of the target public, the DT could also estimate the ideal quantity of vaccines to be available in the immunisation room. In this work, the events of interest will include the temperature measurements collected by sensors and the vaccines administered to patients by dose and type, as recorded in the information systems designed for this purpose.

### 3.2 Architecture based on ISO 23247 - the TwinVax

In this work, the ISO 23247 standard for Digital Twin (DT) frameworks, originally designed for manufacturing, is adapted to a healthcare context. The proposed DT architecture focuses on temperature monitoring through a 2D dashboard and predictive models for anomaly detection and temperature forecasting. It also includes a module for tracking administered vaccine doses by type and dose, enabling cross-referencing with population vaccine needs to support preventive vaccination actions. The DT's functionalities, aligned with ISO 23247, range from monitoring and remote access to simulation, control, optimisation, and predictive analysis, ensuring effective feedback for both users and equipment operations.

**Figure 2:**
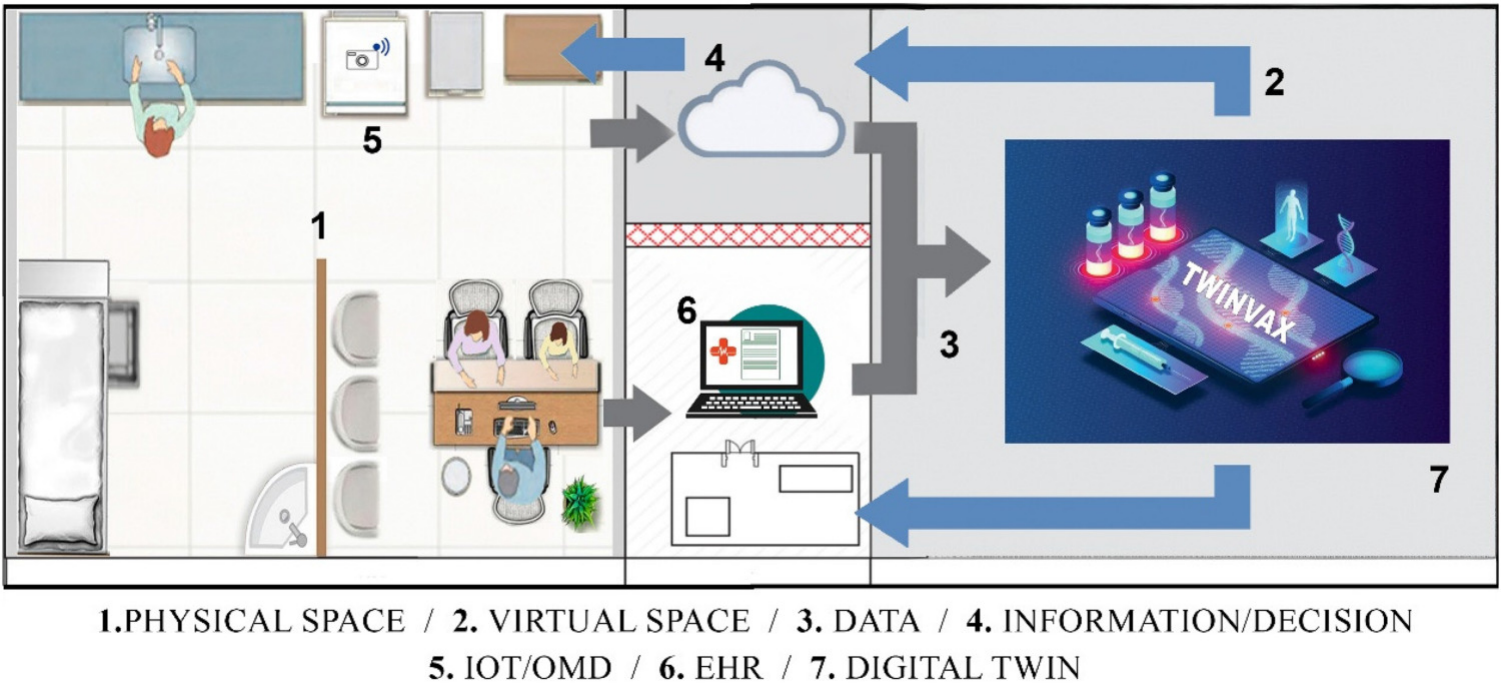
Illustration of the digital twin named TwinVax.

Observing the possibility of integration between IoT architectures and the digital twin modeling proposed in ISO 23247, and adapting them into a simplified and more accessible form for healthcare, this work proposes a four-layer architecture capable of implementing an immunisation digital twin, named TwinVax, as shown in Figure 2. The figure depicts the relationship between the physical and virtual environments within the system. The physical layer includes sensing devices and equipment responsible for real-time data collection, such as temperature sensors and communication modules. These data are transmitted to the virtual environment, where the digital twin processes, stores, and analyzes the information to support simulation, monitoring, and control of the physical system. The communication layer connects these environments, ensuring data integrity, synchronization, and continuous updates. In this way, Figure 2 illustrates the essential link between the real system and its digital counterpart, a concept widely recognized in digital twin research.

The TwinVax architecture is structured into four interconnected domains: Observable Manufacturing Elements (OME), Data Collection and Device Control Entity (DCDCE), Digital Twin Platform (DTP), and User Domain (UD). This layered approach ensures efficient data collection, processing, and utilisation for immunisation management.

#### 3.2.1 Observable Manufacturing Elements (OME)

The Observable Manufacturing Elements (OME) domain corresponds to the physical entities layer, which includes the Ice Lined Refrigerators (ILR) and thermal boxes used for vaccine storage. Within these elements, temperature data will be collected through sensors, which are also integral to the monitoring system. Additionally, the target population for immunisation is considered, with their vaccination data being managed and monitored by the system. To configure the digital twin, information from Electronic Health Records (EHR) including Electronic Medical Records (EMR) and specific Immunisation Information Systems regarding patient data and vaccination records will be integrated. It is important to emphasise that the sensors must adhere to technical standards compatible with industrial communication, such as OPC UA, MQTT, and HTTP, ensuring clear modelling of physical entities and efficient data exchange.

#### 3.2.2 Data Collection and Device Control Entity (DCDCE)

The second layer corresponds to the connection layer, which encompasses the domain of data collection and control of actuating devices present in the previous Observable Manufacturing Elements (OME) layer. This layer facilitates communication and data transfer between the sensors and the digital twin. Data extraction is performed by temperature sensors (e.g., DS18B20, DHT11, or LM35DZ), with initial transformation occurring through local processing, where a device converts electrical signals into data transferable via standard IoT integration protocols.

As the connection layer is based on IoT architecture, temperature sensors will be installed in Ice Lined Refrigerators (ILRs) and thermal boxes to detect variations outside the ideal temperature range, maintained between 2°C and 8°C. These sensors will communicate with the edge layer using the 802.15.4 protocol. Temperature data will be collected once per hour for 5 min, contextualized, and analysed by an algorithm to identify potential issues related to the loss of immunogenic potency due to inadequate storage conditions. Proactive temperature management will be ensured by triggering alerts when temperatures exceed 7°C or fall below 3°C, serving as early warnings that allow preventive actions before reaching critical limits.

The device responsible for transmitting the temperature data to the cloud is the ESP-32 (WROOM-D model), chosen for its strong connectivity features. The ESP-32 is programmed to collect data from the temperature sensors via GPIO pins and transmit this information using Wi-Fi. A server-side application developed in Node.js runs directly on the ESP-32, enabling data processing and transmission via lightweight protocols such as MQTT or HTTP. An MQTT broker receives the temperature data, processes it, and makes it available for authorized subscribers. Additionally, a Node-RED service running on the ESP-32 Gateway acts as an MQTT subscriber, collecting data from the broker and securely transmitting it to the cloud, facilitating real-time monitoring of refrigeration conditions, visualisation of temperature history, and automated notifications in case of deviations.

For enhanced data security, Transport Layer Security (TLS) encryption is applied to all communication between devices, preventing interception or unauthorized modifications. Furthermore, encryption at rest is implemented in storage systems to protect sensitive data.

In addition to the IoT-based monitoring layer, the architecture integrates electronic health records and vaccination registries through standardized interoperability protocols. Data exchange between the Electronic Health Record (EHR), the vaccination registration system, and the digital twin follows the HL7 (Health Level 7) standard, ensuring structured and secure information flow. Both RESTful APIs and GraphQL can be implemented for data synchronization, allowing healthcare professionals to retrieve and manage clinical data and vaccination records efficiently.

Given the critical nature of healthcare data, additional security measures are applied to prevent unauthorized access. OAuth 2.0 authentication and Advanced Encryption Standard (AES) encryption ensure that only authorized users can access and manipulate stored information, safeguarding patient confidentiality. Data transmission is also secured using TLS, aligning with industry best practices and regulatory frameworks such as GDPR.

The entire system infrastructure is hosted in the cloud, ensuring scalability, reliability, and compliance with security standards while allowing seamless integration of IoT data and healthcare information. The choice of cloud provider is flexible and can be adapted based on specific deployment requirements. This combination of IoT monitoring, secure data transmission, and interoperability between healthcare systems ensures an efficient and proactive approach to vaccine storage and management.

#### 3.2.3 Digital Twin Platform (DTP)

At the core of TwinVax, the Digital Twin Platform (DTP) layer is responsible for managing application services, data processing, and system operations. This layer supports critical functions such as data analysis, aggregation, rule application, and storage, ensuring the seamless operation of the TwinVax system. To achieve robust performance and scalability, the architecture leverages Amazon Web Services (AWS), utilizing services like AWS IoT SiteWise and Node-RED for efficient data processing, transformation, and presentation.

Communication within the DTP is streamlined using the MQTT protocol, which optimizes IoT data transmission by reducing the computational load on devices, especially low-power ones such as the ESP-32. This efficiency is crucial for maintaining real-time responsiveness while conserving device resources. The data transformation process involves converting raw data collected from sensors and health records into actionable metrics. This includes calculating average daily temperatures and assessing vaccination coverage rates across different population segments, providing valuable insights for immunisation management.

For data storage, the DTP employs time-series databases like InfluxDB or TimescaleDB. These databases are specifically designed to handle continuous monitoring data efficiently, supporting the long-term management and analysis of large volumes of time-stamped data generated by vaccine storage monitoring and immunisation tracking.

Scalability is a fundamental consideration for TwinVax, particularly when expanding to larger healthcare networks or national health systems. To support this, the architecture adopts a microservices approach, allowing individual system components—such as temperature monitoring, vaccine management, and patient records—to be updated, scaled, and maintained independently. This modularity enhances system flexibility and simplifies maintenance without disrupting overall operations. Additionally, the elasticity of cloud services enables dynamic resource allocation, automatically adjusting computing and storage capacity to meet fluctuating data demands. In scenarios with high data traffic, the use of content distribution networks (CDNs) and load balancing mechanisms ensures efficient data delivery, system reliability, and high availability, even under peak load conditions.

#### 3.2.4 User Domain (UD)

This layer is designed to support healthcare professionals and managers by providing access to data visualisation and decision-support tools. It includes services such as temperature monitoring dashboards, predictive vaccination analyses, and automated notifications. The data visualisation aspect relies on interactive dashboards, such as Grafana, which display real-time information with colour-coded indicators—green for normal conditions, yellow for alerts, and red for emergencies. Historical data trends are also available, enabling quick and clear monitoring of the situation.

Additionally, predictive analyses help forecast vaccine demand and identify individuals who are due for immunisation, supporting proactive planning. To ensure timely communication, the system includes SMS alerts for staff and patients, push notifications through mobile apps, and on-site visual alarms. These elements together create a comprehensive architecture, allowing for effective vaccine management by enhancing real-time monitoring, forecasting, and data-driven decision-making in immunisation programs.

## 4 Results

**Figure 3:**
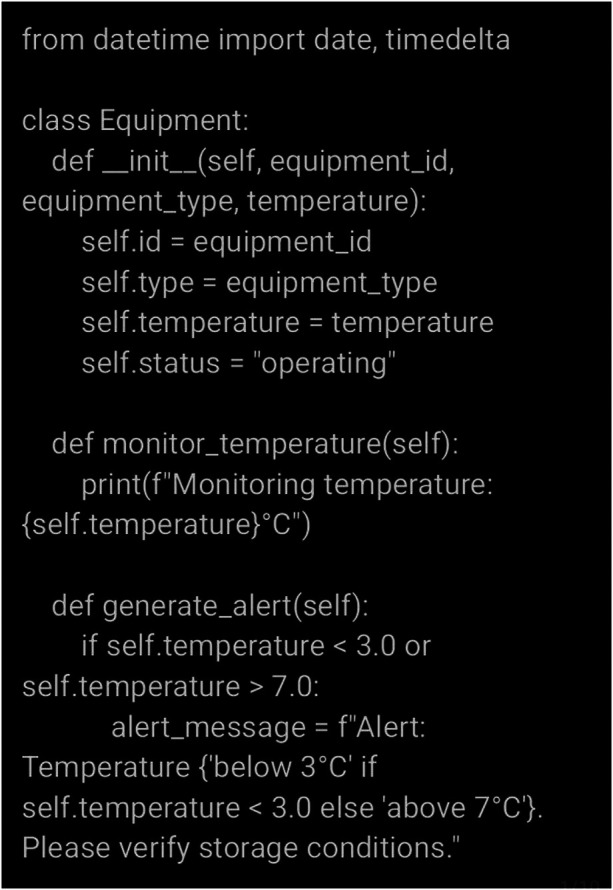
Portion of the code for simulation.

To demonstrate the practical applicability of TwinVax, based on the proposed modelling and architecture, an environment for simulating discrete events using Python - SimPy - was employed. SimPy allows for modelling complex systems involving concurrent processes, such as queues, waiting times, and interactions between different entities over time. In this study, SimPy was used to simulate the operation of TwinVax, enabling the evaluation of system behaviour under various conditions, such as temperature variations in storage equipment and fluctuations in vaccination coverage. This validation step ensures that temperature alerts and real-time vaccination coverage analyses function as expected before actual implementation. A portion of the code for the simulation is described in Figure 3:

The scenarios considered in the simulation encompassed typical and adverse operational conditions, such as stable temperature maintenance within the recommended 2°C–8°C range, abrupt temperature deviations caused by equipment malfunctions, and intermittent network connectivity affecting data transmission. Key parameters included hourly temperature measurements lasting five minutes, alert thresholds set at 3°C (minimum) and 7°C (maximum), and data buffering using FIFO queues for temporary local storage.

**Figure 4:**
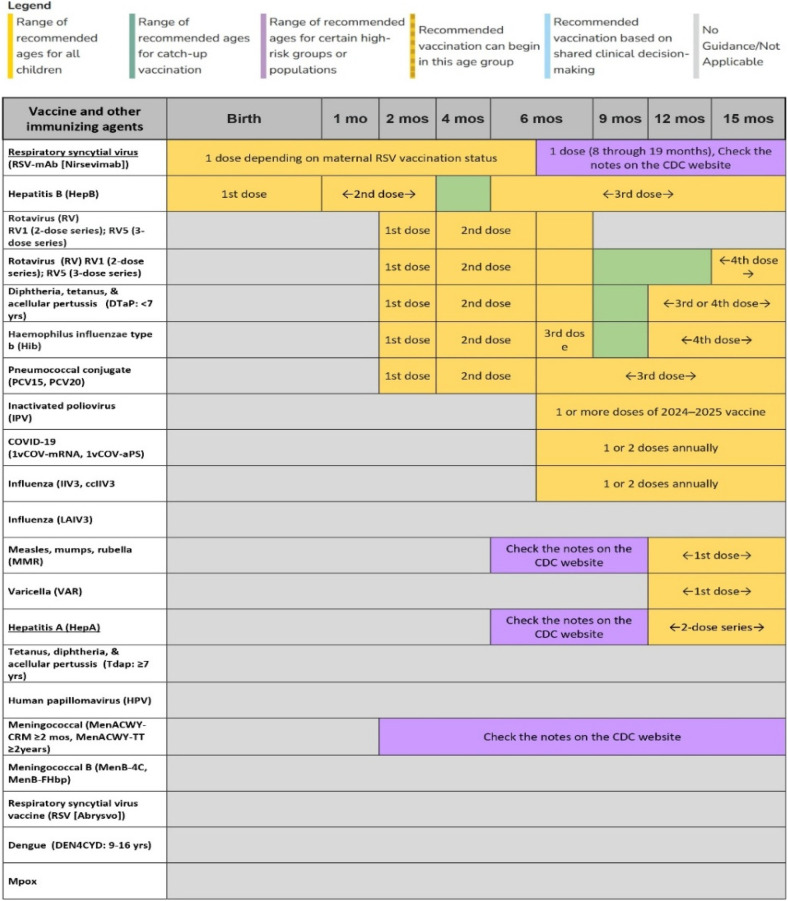
Example of vaccination schedule for children under 15 mouths - WHO.

For this study, the WHO vaccination schedule for 2024 (Figure 4) was used, focusing on a population of 3,527 residents of Rio de Janeiro, aged between 1 and 90 years. To ensure representativeness and robustness, stratified sampling was employed to capture the age distribution aligned with vaccination schedules. A random sample of 100 individuals was selected, proportional to each age group, satisfying a 95% confidence level and a 10% margin of error. The following groups were considered: under 1 year (3 individuals), 1–6 years (7), 7–9 years (3), 10–19 years (8), 20–39 years (23), 40–59 years (28), and 60+ years (28).

Specifically, the age groups were selected to represent the distinct vaccination schedules for infants, children, adults, and the elderly, each with different vaccination requirements and intervals. This stratification allows the system to simulate varying vaccination coverage and test TwinVax's ability to manage vaccines across different stages of life.

To validate the simulation, a basic statistical analysis was conducted, including the calculation of means, standard deviations, and confidence intervals for key indicators such as temperature stability and alert response times. Additionally, the simulated outputs were compared with real-world data and established vaccination guidelines to ensure consistency and model accuracy. This validation step reinforces the credibility of the system's predictive capabilities and operational readiness.

The TwinVax Digital Twin is designed to ensure both the optimal storage conditions of vaccines and the timely administration of vaccines to patients. The system achieves this by continuously monitoring vaccine storage temperatures and tracking vaccination schedules. The operational flow is outlined as follows:

### 4.1 Equipment monitoring

Refrigerators and thermal boxes are equipped with temperature sensors that continuously monitor and transmit the temperature. When the temperature falls below 3°C or exceeds 7°C, the system triggers an alert to prevent vaccine spoilage. Alerts are sent via SMS and WhatsApp to key stakeholders: the immunisation team, health centre manager, and local health authority. The alert message follows a standardized format:

“Alert: Temperature below 3°C or above 7°C. Please verify storage conditions.”

### 4.2 Vaccine monitoring

Each vaccine is identified by its name (e.g., BCG, Hepatitis B) and type (e.g., routine or campaign). The system tracks the required number of doses and ensures that vaccination deadlines are met.

### 4.3 Patient tracking

Each patient profile includes their name, date of birth, and a list of vaccines they need to receive. The system monitors both administered and pending vaccines, ensuring that no dose is missed. In addition, vaccination alerts are generated for patients and directed to both the patient and the healthcare team, including the health unit manager. These alerts follow the format:

“Vaccination Alert: Patient (patient's name) must receive the (vaccine name) within (X number of days).”

The number of days for the alert will be determined based on the patient's age group. For those under 1 year old, alerts are set for 7, 15, and 30 days prior to the vaccination deadline. For individuals over 1 year old, alerts are set for 30, 60, and 90 days before the expected vaccination date.

### 4.4 Digital Twin functionality

The Digital Twin serves as the core of the system, functioning as the “brain” that continuously monitors both temperature and vaccination schedules. In the event of temperature deviations or approaching vaccination deadlines, the Digital Twin sends alerts to the appropriate parties, prompting immediate action.

#### 4.4.1 Example 1 (paediatric patient: Alice)

Consider Alice, a one-month-old infant, who is scheduled to receive the BCG vaccine within 30 days of birth. The TwinVax system is monitoring the refrigerator that stores the BCG vaccine. If the temperature of the refrigerator falls to 2.8°C, the system immediately triggers an alert:

“Alert: Temperature below 3°C. Please verify storage conditions.”

This alert is sent via SMS and WhatsApp to the immunisation team, the unit manager, and the local responsible party. Simultaneously, TwinVax identifies that Alice's vaccination deadline is approaching. As she must receive the BCG vaccine within the next 15 days, the system sends a second alert:

“Vaccination Alert: Alice must receive the BCG vaccine within 15 days!”

Both alerts are triggered simultaneously: the first relates to the preservation of the vaccine's integrity, while the second ensures the timely administration of the vaccination. If the refrigerator temperature remains outside the ideal range (below 3°C), the team can act swiftly, ensuring the vaccine remains effective until it is administered. Given Alice's age, the vaccination alert is sent to her legal guardian (the responsible party) as she is a minor, as well as to the local healthcare team, accompanied by a suggested date for vaccination.

#### 4.4.2 Example 2 (adult patient: John)

Now, consider John, a 35-year-old adult, who is due to receive the tetanus vaccine. According to the vaccination schedule, John must receive the vaccine within the next 60 days. TwinVax is monitoring the refrigerator storing the tetanus vaccine. If the temperature of the refrigerator rises to 7.1°C, the system immediately triggers an alert:

“Alert: Temperature above 7°C. Please verify storage conditions.”

This alert is sent via SMS and WhatsApp to the immunisation team, the unit manager, and the local responsible party. Simultaneously, TwinVax verifies that John's vaccination deadline is nearing. As he must receive the tetanus vaccine within 60 days, the system also sends a specific vaccination alert:

“Vaccination Alert: John must receive the tetanus vaccine within 60 days!”

These simultaneous alerts help ensure both the quality and safety of the vaccine, as well as serve as a reminder to the team and John regarding the vaccination schedule, preventing the dose from being missed or administered incorrectly. Since John is an adult, the vaccination alert is sent directly to him, as well as to the local healthcare team, again with a suggested vaccination date.

In both examples, it is essential to note that:

Temperature Alerts: The system detects any variation outside the optimal temperature range (3°C–7°C), triggering immediate alerts to the responsible parties. This enables rapid corrective actions, such as verifying the functionality of the refrigerator or relocating the vaccine to another unit, ensuring the vaccine's preservation.

Vaccination Alerts: The system monitors the vaccination schedules of patients, sending alerts as the vaccination date approaches. In the case of minors, the alert is directed to the legal guardian (e.g., Alice's parent), while for adults, it is sent to the patient (e.g., John). In both cases, the alert is also sent to the local healthcare team, accompanied by a suggested vaccination date. For Alice, the suggested vaccination date is 15 days prior to the deadline, while for John, it is 60 days prior to his vaccination date.

Immediate Actions: The responsible parties can take immediate actions based on the alerts, including adjusting temperature settings or scheduling the vaccination appointment as required. The multi-alert mechanism ensures a prompt and effective response, preventing errors in vaccine administration and ensuring their quality.

Based on these examples, it was possible to verify that the TwinVax system effectively ensured both vaccine integrity and timely vaccination. The system prevented temperature deviations while issuing alerts that ensured on-time vaccinations. This simulation confirmed the TwinVax system's reliability in real-world conditions, validating its ability to support successful immunisation programs.

## 5 Discussion

TwinVax was developed as a solution for the rigorous management of thermal conditions in the storage of immunobiologicals within primary healthcare immunisation services. The system collects, stores, monitors, and visualises temperature data from sensors installed in Ice Lined Refrigerators (ILRs) and thermal boxes, while also tracking vaccination coverage through data extracted from information systems related to vaccine administration and vaccinated individuals.

In designing TwinVax, several critical factors were taken into account. Data collection involves continuous temperature measurements, electronic health records (EHR), vaccination history, vaccine types, and administration dates. Temperature data are gathered hourly for five minutes, whereas EHR data are collected at the end of each working day, with the possibility of adjusting this frequency based on the healthcare team's needs. In critical situations, data collection frequency may increase to provide more detailed real-time insights.

Data acquisition is performed through sensors connected to an ESP-32 device, which uses communication protocols such as MQTT or HTTP for data transfer. In cases of network unavailability, the ESP-32 temporarily stores data locally, for example in a SQLite database, employing a FIFO (first-in, first-out) buffer to maintain record sequencing. Data transmission to the cloud can operate in hybrid mode, using periodic batch uploads.

Preferred data storage occurs in the cloud, utilising scalable databases such as InfluxDB or TimescaleDB for ease of access. When connectivity is compromised, local storage is employed. The data are organised to allow streamlined access, adhering to retention and cleaning policies to ensure integrity and compliance.

Actions within the immunisation service rely on analyses conducted in the digital twin environment. Physical interventions may be necessary in critical cases, such as sudden temperature fluctuations, with real-time notifications sent to healthcare teams via SMS and WhatsApp. Vaccination coverage, a key indicator of programme performance, is assessed through registries, routine reports, and household surveys. Effective monitoring enables identification of individuals requiring immunisation, facilitating timely and informed decision-making. Furthermore, automated alerts remind individuals of upcoming vaccination deadlines, improving adherence to immunisation schedules.

TwinVax employs interactive dashboards, such as Grafana, to provide real-time visualisation of storage conditions and vaccination coverage. Historical data—including temperature logs, vaccination records, and demographic information—are stored for analysis and reporting. Visualisation tools include temperature graphs, alert indicators, and coverage data, providing clear real-time status updates.

The system identifies individuals approaching their vaccination dates and generates timely alerts to improve adherence. Integration with EHRs enables proactive communication through SMS reminders, reinforcing compliance with vaccination schedules. Additionally, TwinVax supports predictive analyses, such as forecasting vaccine demand and detecting storage equipment with recurring temperature deviations. Machine learning models—including decision trees and Support Vector Machines (SVMs)—may enhance these capabilities by analysing historical trends and environmental factors. For healthcare professionals, TwinVax offers an intuitive interface for real-time monitoring of storage conditions and vaccination coverage, supporting evidence-based decision-making regarding temperature control and immunisation strategies.

By addressing these critical factors and providing concrete solutions, TwinVax presents itself as a fully viable approach grounded in continuous monitoring of essential vaccine conservation conditions, combined with optimisation of vaccination interventions according to the current schedule. The system integrates advanced anomaly detection and time-series prediction technologies, fostering data-driven management to guarantee the safety and efficacy of stored immunobiologicals.

Strict temperature control within ILRs and thermal boxes forms one of the system's fundamental pillars. Minor temperature variations may jeopardise the immunogenic capacity of vaccines, causing significant losses. To mitigate such risks, TwinVax employs high-precision sensors to ensure early detection of critical deviations, reducing losses and preserving immunobiological integrity. Another distinguishing feature is its predictive module for analysing vaccination coverage. This functionality enables health teams to anticipate demand and optimise actions, minimising the need for active outreach to individuals with delayed vaccinations. Real-time cross-referencing of administered doses with vaccination calendar schedules enables early immunisation interventions, ensuring that the right people are vaccinated at optimal times, thereby promoting greater adherence and coverage. Automated alerts for health professionals facilitate prioritisation of personalised actions, especially targeting vulnerable populations or regions. Consequently, immunisation campaigns become more impactful, enhancing public health protection.

A key prospective functionality to enhance TwinVax is risk analysis for non-compliance with vaccination schedules based on socio-economic and demographic variables. Such factors—including maternal education, family income, APGAR scores at birth, birth complications, and place of residence—would be processed and weighted by machine learning algorithms. These models would predict the likelihood of individuals failing to adhere to vaccination schedules, enabling targeted interventions.

Machine learning techniques such as decision trees, Support Vector Machines, random forests, and gradient boosting could be used to develop this predictive model. The system would continuously refine its predictions by incorporating real-time health data, vaccination history, and socio-economic indicators, allowing the digital twin to provide timely and accurate risk assessments.

Cloud-based AI platforms would facilitate secure data processing, ensuring compliance with privacy regulations such as GDPR while supporting model scalability. Beyond monitoring vaccination status, the predictive model would assist in designing personalised interventions, including reminder messages and outreach by health educators, thereby improving vaccination uptake.

These algorithms rely on key predictors such as antenatal care visits, institutional deliveries, maternal health, and socio-economic status. By focusing on these elements, the model can identify individuals at higher risk of vaccination non-compliance, facilitating more effective resource allocation and increasing vaccination rates while reducing preventable diseases.

Though not a primary focus during the initial development phase, TwinVax holds potential for optimising vaccine stocks. By correlating predicted demand with current inventory, the system could reduce waste and prevent shortages of immunobiologicals, reinforcing its role as a comprehensive and strategic immunisation management tool.

Another important aspect of TwinVax is its contribution to improving patient experience. Integration with electronic health records enables proactive communication through SMS alerts reminding patients of vaccination dates. This direct engagement may increase adherence, especially in regions with high rates of forgetfulness or resistance. For health professionals, the platform offers efficient real-time monitoring via interactive dashboards such as Grafana, fostering evidence-based decisions in temperature management and vaccination effectiveness. Clear visual alerts and critical indicators facilitate swift corrective actions, minimising delays in responding to risk situations. Thus, TwinVax provides health workers with accurate, timely information essential for effective service management and epidemiological challenges.

The adoption of TwinVax enables significant advances in coordination and governance of immunisation efforts. Its predictive capabilities improve resource management and provide a solid foundation for implementing targeted and effective public health policies. Historical data analysis allows vaccination strategies to be tailored to local needs, ensuring more effective coverage and better population health outcomes. Hence, TwinVax emerges as a robust, innovative solution for modernising primary healthcare processes. Further evaluation of its strategic and operational benefits for both healthcare professionals and populations served remains an important future step.

Despite these advantages, certain limitations affect TwinVax implementation. Integrating diverse data systems—such as EHRs and IoT temperature sensors—poses technical challenges due to device heterogeneity and varying standards across healthcare units, despite protocols like HL7 and MQTT. Connectivity issues and limited bandwidth, especially in remote areas, may cause delays in real-time data collection and alert generation. Furthermore, delayed or absent vaccination records hamper the digital twin's ability to mirror current operational states accurately. Such latency can undermine vaccine cold chain monitoring, risking compromised efficacy. Elevated response delays weaken the system's reliability as a real-time support tool.

Another challenge lies in modelling the dynamic operational environment of immunisation services, which are highly context-sensitive and frequently changing due to updated protocols, demand fluctuations, outreach campaigns, and logistics disruptions. Static digital models may quickly become outdated, reducing analytical value and decision support. Maintaining model accuracy demands ongoing recalibration of process flows, thresholds, and logic, requiring domain expertise and increasing operational complexity and costs.

Moreover, adapting an architecture originally developed for manufacturing (ISO 23247) to the public health context necessitates simplifying manufacturing-specific terminology into accessible language suitable for immunisation and vaccine monitoring. Ensuring data security and patient privacy remains paramount, requiring compliance with strict regulations such as GDPR.

Training healthcare staff to fully utilise TwinVax also presents challenges. Implementing new technology involves technical integration and a learning curve for daily users. While the dashboard's user-friendly interface and automated notifications mitigate this, adaptation may be slow in areas with limited digital literacy.

Sustainability and scalability are critical considerations. Expanding TwinVax to more complex scenarios—different vaccines and logistic conditions—requires financial and technological resources. The costs of IoT infrastructure, cloud storage, and ongoing maintenance must be carefully weighed by health authorities before broader deployment. Nonetheless, TwinVax demonstrates valuable potential in enhancing immunisation through proactive monitoring and increasing vaccination coverage.

Governance and compliance are essential for implementation due to the sensitive nature of health data. TwinVax ensures data privacy through encryption and strict access controls, complying with regulations such as GDPR. The architecture includes audit mechanisms to trace and validate system actions, such as alert issuance and automated decisions, ensuring interventions align with public health policies and ethical standards.

Data storage and management adhere to the highest security standards, restricting access to authorised users only. Interactions with external health systems like EHRs occur via secure, auditable APIs, maintaining transparency and security as required by regulatory bodies.

Beyond privacy, accurate interpretation of digital twin data is crucial for vaccination strategy effectiveness. Continuous temperature monitoring maintains vaccine integrity, preventing compromises that would affect efficacy. The system's true impact lies in enabling health professionals to make agile, informed decisions, anticipating risks such as patient no-shows due to forgetfulness, disinterest, or family dynamics. Accurate predictions allow proactive, targeted interventions, improving vaccination coverage and timely protection.

Finally, ethical considerations cannot be overlooked. TwinVax must serve all population groups equitably, avoiding discrimination in vaccine access. Automated decision-making—such as targeting campaigns based on coverage forecasts—requires human oversight to incorporate social and cultural factors. Transparency in operations and clear communication about data use are vital to building trust and ensuring effective public health contributions.

## 6 Conclusions

TwinVax offers an innovative approach to immunisation services within primary healthcare by combining real-time temperature monitoring with vaccination coverage tracking. Its integration of IoT devices, cloud storage, and advanced analytics ensures strict control over the thermal conditions essential for vaccine preservation, while supporting timely, data-driven decisions in immunisation management.

By continuously gathering data from sensors and electronic health records, TwinVax enables quick responses to critical situations such as temperature deviations through real-time alerts and interactive dashboards. Its predictive features, powered by machine learning models, further extend its impact by forecasting vaccine demand, identifying individuals at risk of missing vaccinations, and optimising resource allocation.

Implementing TwinVax represents an important step forward in public health, especially in maintaining vaccine effectiveness and reducing waste. By setting minimum standards for storage and allowing scenario analyses to identify vulnerable groups, it promotes a more strategic, evidence-based management of immunisation, ensuring vaccines are used safely and responsibly, benefiting both patients and healthcare systems.

A key strength of TwinVax is its originality: it proposes a digital twin tailored specifically for immunisation services, integrating both cold chain monitoring and electronic health record data. This combined approach is particularly valuable for low- and middle-income countries, where vaccination coverage and logistics face significant challenges. TwinVax has the potential to serve as a reference model for national programs aiming to modernise their operations through digital technologies.

We also acknowledge some limitations. Validation so far has been based on simulations, which limits the direct applicability of the results without real-world field testing. Connectivity issues in remote areas may affect continuous data transmission, and integrating diverse health information systems may pose technical challenges that need future solutions.

Despite these challenges, TwinVax shows resilience through secure encryption protocols, strict access controls, and auditing mechanisms that protect data integrity. Its flexibility to operate in various environments, both locally and in the cloud, reinforces its potential for scalability and sustainability.

Ethical concerns related to automated decision-making are addressed through human oversight and transparent governance, promoting fairness in access and public trust. Moreover, TwinVax's architecture allows the integration of additional modules, facilitating adaptation to future technological advances and expanding its applicability across healthcare domains.

In summary, TwinVax demonstrates how digital twin technology can transform immunisation services. By enhancing vaccine safety, reducing waste, and supporting proactive health management, it offers a robust and scalable solution aligned with the ever-changing needs of health systems.

## Data Availability

The data analyzed in this study is subject to the following licenses/restrictions: the data utilised in this study originate from the database of a population registered within a health region in the state of Rio de Janeiro. In accordance with the prevailing legislation, these data are protected under privacy and security regulations, including the General Data Protection Law (LGPD – Law No. 13,709/2018). The LGPD establishes strict guidelines for the processing of personal data, requiring safeguards against unauthorised access, breaches, and improper use. Furthermore, the use of these data must adhere to the principles of necessity, purpose, and data minimisation, ensuring that only the information strictly required for the research is accessed and analysed. Any sharing or processing of the data must be conducted on the basis of informed consent or other applicable legal grounds, as stipulated by regulatory bodies and health authorities. Accordingly, this study fully complies with ethical and legal standards to ensure the privacy and security of individuals whose data are being analysed. Requests to access these datasets should be directed to vh2005dias@gmail.com.
